# Supplementary material for: Growth Potential of Listeria monocytogenes on Refrigerated Spinach and Rocket Leaves in Modified Atmosphere Packaging
Source: Foods. 2020 Sep 1;9(9):1211. doi: 10.3390/foods9091211 (PMC7555703; doi:10.3390/foods9091211)
Supplement: Supplementary file 1 [file foods-09-01211-s001.pdf]

Supplementary Materials - Supplementary tables and figures

Table 1. ComBase Data based on data over 7 days.

Product	Inoculation Density CFU g^{-1}	Linear Model R2	Linear Model RMSE	Linear model Maximum Growth Rate log CFU day^{-1}
**Spinach**	100	0.978	0.134	0.290
**Spinach**	100	0.975	0.185	0.370
**Spinach**	100	0.959	0.251	0.394
**Spinach**	10	0.995	0.073	0.336
**Rocket**	100	0.985	0.091	0.235
**Rocket**	100	0.931	0.197	0.237
**Rocket**	100	0.841	0.175	0.133
**Rocket**	10	0.929	0.241	0.288
**Lettuce**	100	0.945	0.143	0.193
**Lettuce**	100	0.998	0.058	0.377
**Lettuce**	100	0.959	0.151	0.235
**Lettuce**	10	0.936	0.173	0.215

Table 2. ComBase Data based on data over 9 days.

	Inoculation Density CFU g^{-1}	Linear Model R^2	Baranyi and Roberts Model R^2	Linear Model RMSE	Baranyi and Roberts Model RMSE	Linear Model Maximum Growth Rate log CFU day^{-1}	Baranyi and Roberts Model Maximum Growth Rate log CFU day^{-1}
**Spinach**	100	0.974	0.977	0.159	0.148	0.266	0.293
**Spinach**	100	0.956	0.986	0.253	0.145	0.326	0.401
**Spinach**	100	0.926	0.986	0.342	0.148	0.336	0.557
**Spinach**	10	0.997	0.996	0.064	0.077	0.329	0.334
**Rocket**	100	0.968	0.983	0.140	0.102	0.210	0.240
**Rocket**	100	0.958	0.940	0.170	0.203	0.225	0.235
**Rocket**	100	0.852	0.981	0.274	0.097	0.184	0.346
**Rocket**	10	0.964	0.975	0.184	0.154	0.262	0.305

Table 3. Total heterotrophic bacteria in ready-to-eat salad vegetables [log CFU g^{-1}] ± standard. deviations. Different letters indicate significant differences.

Product	Day 0	Day 9 (spinach and rocket) / Day 7 (lettuce)
**Spinach**	6.96^{A} ± 0.066	8.86^{B} ± 0.150
**Rocket**	5.94^{a} ± 0.376	7.97^{b} ± 0.328
**Lettuce**	7.11^{α} ± 0.369	8.69^{β} ± 0.156

Table 4. Water Activity and pH values ± standard deviations.

	Spinach Water Activity	Ph Spinach	Rocket Water Activity	Rocket pH	Lettuce Water Activity	pH lettuce
**Day 0**	0.974 ± 0.001	7.30 ± 0.078	0.980 ± 0.002	6.55 ± 0.153	0.993 ± 0.001	6.34 ± 0.029
**Day 2**	0.978 ± 0.002	7.21 ± 0.056	0.981 ± 0.005	6.46 ± 0.108	0.988 ± 0.003	6.40 ± 0.249
**Day 5**	0.972 ± 0.003	6.93 ± 0.163	0.979 ± 0.001	6.75 ± 0.046	0.996 ± 0.002	6.25 ± 0.062
**Day 7**	0.974 ± 0.001	7.12 ± 0.059	0.978 ± 0.004	6.60 ± 0.090	0.991 ± 0.005	6.36 ± 0.142
**Day 9**	0.970 ± 0.002	7.25 ± 0.038	0.976 ± 0.001	6.86 ± 0.086		

Table 5. Oxygen concentrations (%).

Product	Inoculation Density CFU g-1)	Day 0 (oxygen concentration %)	Day 7 (oxygen concentration %)	Day 9 (oxygen concentration %)
**Spinach**	100	4.00	9.18	10.03
**Spinach**	100	4.08	10.70	10.93
**Spinach**	100	4.10	10.32	10.82
**Spinach**	10	4.15	11.73	10.80
**Rocket**	100	4.00	9.20	10.48
**Rocket**	100	4.03	9.48	10.33
**Rocket**	100	4.00	9.89	10.41
**Rocket**	10	4.05	9.35	10.40
**Lettuce**	100	4.10	10.23	
**Lettuce**	100	4.03	9.31	
**Lettuce**	100	4.01	10.10	
**Lettuce**	10	4.05	9.58	

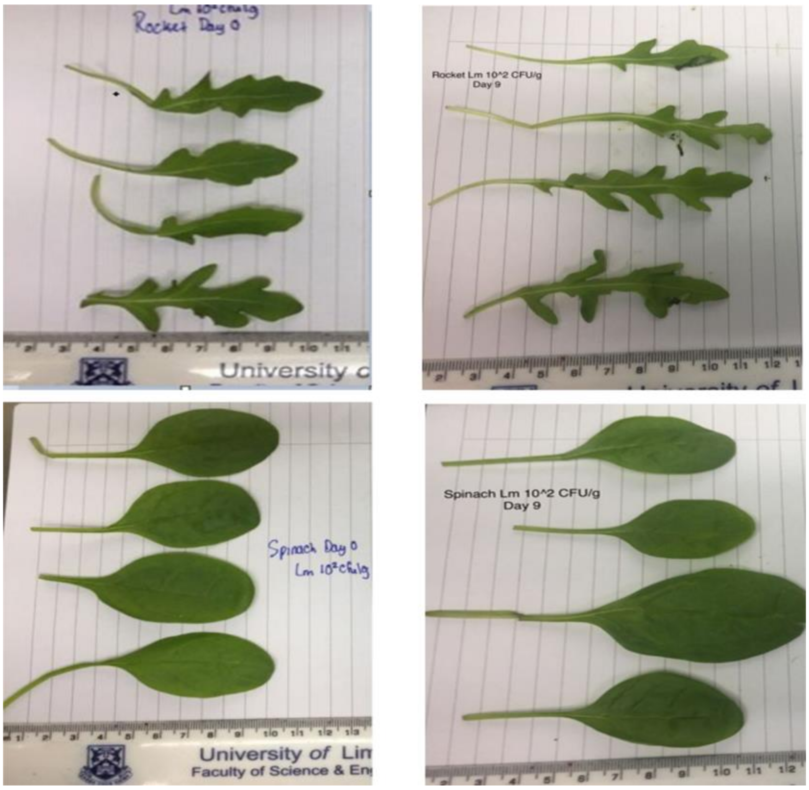

Figure 1. Images of rocket (day 0, 100 cfu g^{-1}, top left), rocket (day 9, 100 cfu g^{-1}, top right), spinach (day 0, 100 cfu g^{-1} bottom left) and spinach (day 9, 100 cfu g^{-1}, bottom right) for visual appearance analysis.
